# Rapid diagnostic testing combined with an immediate infectious disease consultation increases the rate of septic intensive care unit patients on targeted antibiotic therapy

**DOI:** 10.3389/fcimb.2024.1513408

**Published:** 2025-01-21

**Authors:** Evelyn Kramme, Nadja Käding, Tobias Graf, Karolin Schmoll, Heidi Linnen, Katharina Nagel, Esther Grote-Levi, Susanne Hauswaldt, Dennis Nurjadi, Jan Rupp

**Affiliations:** ^1^ Department of Infectious Diseases, University of Lübeck and University Hospital Schleswig-Holstein, Lübeck, Germany; ^2^ Institute of Medical Microbiology, University of Lübeck and University Hospital Schleswig-Holstein, Lübeck, Germany; ^3^ German Center for Infection Research, Hamburg-Lübeck-Borstel-Riems site, Lübeck, Germany; ^4^ University Heart Center Lübeck and German Center for Cardiovascular Research, University Hospital Schleswig-Holstein, Lübeck, Germany; ^5^ Department of Anaesthesiology, University of Lübeck and University Hospital Schleswig-Holstein, Lübeck, Germany

**Keywords:** sepsis, rapid diagnostic testing, infectious diseases consultation, adequate antibiotic therapy, blood stream infection (BSI), antimicrobial stewardship (AMS)

## Abstract

**Objectives:**

To evaluate the impact of rapid diagnostic testing (RDT) combined with immediate infectious disease (ID) consultation on the treatment of septic patients with positive blood cultures in intensive care units in a setting without 24/7 service.

**Methods:**

Adult ICU patients in a tertiary care hospital with positive blood cultures were included from January 2019 to December 2020. The control group underwent routine laboratory testing, and for the intervention group, RDT was applied with immediate ID consultation.

**Results:**

In 77 out of the 91 patients in the intervention group, the pathogen was identified by RDT. Regarding antimicrobial susceptibility testing (AST), genotypic testing (ePlex^®^) was successful for Gram-positive cocci, but inadequate for Gram-negative rods. Phenotypic resistance testing with the Accelerate PhenoTest^®^ took too long to be successfully integrated into the intervention. Adaptation of empirical antibiotic therapy was recommended for 72.7% of the patients. Adherence to the ID consultation post-RDT results was high at 82.3%. In the control group, adaptation of the initial antibiotic therapy would have been recommended for 81.8% of patients, if the species identification had been available. Overall adherence to the local antibiotic therapy guideline for sepsis was significantly lower in the control than in the intervention group (27.8% versus 89.3%, p<0.001).

**Conclusion:**

Integration of an RDT system in the microbiological workflow for septic patients in ICU combined with a standardized ID intervention led to a significantly higher percentage of adequate antimicrobial treatment and greater adherence to local antibiotic therapy recommendations, even in a setting where 24/7 service is not available.

## Introduction

Sepsis is associated with high morbidity and mortality. Every hour of treatment delay decreases the survival rate by 7.6% (Kumar et al., 2006). The need for early appropriate antimicrobial therapy leads to the use of broad-spectrum antibiotics. A commitment to de-escalation is essential to reduce side effects in patients (Niederman et al., 2021) (e.g., microbiome, *Clostridioides difficile* infection) (Ravi et al., 2019) and the development of resistance. Early adaptation to targeted therapy is facilitated by rapid pathogen identification and timely infectious disease (ID) counseling (Neuner et al., 2016) in patients with sepsis.

Molecular analysis techniques, representing a combination of bacterial culture and molecular diagnostics, are on the rise (Banerjee et al., 2015). Rapid molecular identification of pathogens (< 1.5 h) and accelerated phenotypic antimicrobial susceptibility testing (AST) (< 7 h) led to comparable results to culture methods in 24-36 h. The cost-effectiveness of rapid diagnostics has been shown for bloodstream infections (BSIs) in combination with antimicrobial stewardship (AMS) (Pliakos et al., 2018). In both Gram-positive and Gram-negative BSIs, the introduction of rapid diagnostic tests (RDTs) and AMS reduced the time to targeted antibiotic therapy (Beal et al., 2015).

In Gram-negative BSIs, the proportion of optimally treated patients increased, especially among those who received ID consultations (Claeys et al., 2020). Yet 24/7 laboratory service and ID consultation are rarely available in German hospitals. Evidence supporting the clinical impact of rapid identification and AST is limited and controversial for sepsis patients with bacteremia.

The aim of this study was to evaluate the integration of an RDT system into the microbiological workflow for septic patients with blood culture results. Our goal was the timely achievement of targeted therapy to improve treatment outcomes and avoid the collateral damage of a broad-spectrum therapy even though no 24/7 service was available.

## Materials and methods

### Study design

**Figure 1:**
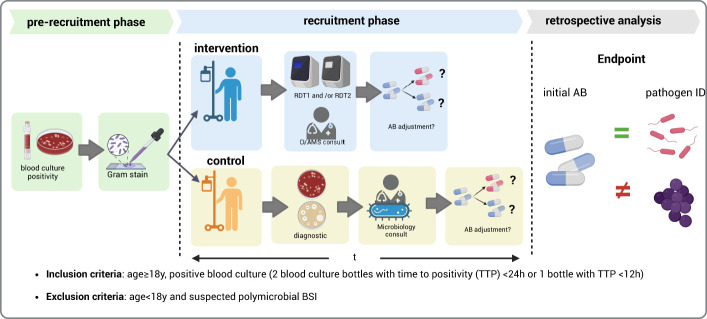
Overview of study design. Comparison of rapid diagnostic testing (RDT) using ePlex® (RDT1) and Accelerate® (RDT2) on the time to effective therapy as defined by the pathogen spectrum covered by the initial antibiotic therapy. (Figure created with Biorender.

We conducted a prospective cohort study of patients with positive blood cultures and sepsis in two ICUs in the tertiary care University Hospital of Schleswig-Holstein in Lübeck between August 2018 and December 2020. In the intervention group patients’ blood cultures underwent RDT and immediate ID consultation. The standard of care group underwent conventional microbiological diagnostics (**Figure 1**). The study was approved by the local ethics committee (University of Lübeck, Az 18-261).

Microbiological, clinical, laboratory, and treatment-related data were collected from the index blood cultures. Patients were stratified into the intervention and standard of care study arms upon the detection of blood culture positivity during routine microbiological diagnostics. In the intervention group, RDT was conducted using ePlex^®^ and Accelerate^®^ on the basis of Gram staining. The results were conveyed to the attending physician by the infectious disease/antimicrobial stewardship (ID/AMS) consult team. If required according to the RDT results, adjustments to antibiotic (AB) therapy were recommended (**Supplementary Table S1**). In the standard of care group, the microbiology specialist reported the Gram staining results to the attending physician and suggested antibiotic adjustments. Adherence to the recommendations for AB adjustment was assessed upon completion of the study through a review of patient charts and microbiology results (**Figure 1**).

### Microbiology

#### Conventional blood culture

Routine laboratory diagnostic procedures were conducted according to local microbiology laboratory protocols using the BacT/Alert (bioMérieux, Nürtingen, Germany) automated system. The results of the Gram stains were immediately communicated to the attending physicians for both the control and intervention group by the clinical microbiology specialist. Species identification was performed using MALDI-TOF mass spectrometry (Bruker Daltonics, Germany). AST was performed using a VITEK^®^2 (bioMérieux, Germany) and was interpreted according to the valid EUCAST clinical breakpoints of the respective year (www.eucast.org/ast_of_bacteria v 8.1, v 9.0, v 10.0).

#### Rapid diagnostic testing

Rapid diagnostic for species identification and AST was performed with the Accelerate^®^ PhenoTest™ BC kit (Accelerate Diagnostic, Tucson, Arizona) and the ePlex^®^ system (GenMark Diagnostics, Carlsbad, CA) according to the manufacturer’s instructions. The Accelerate Pheno^®^ system is based on microbial identification by fluorescence *in situ* hybridization (FISH) combined with AST assessments (BC kit), while ePlex^®^ is based on a multiplex microarray assay. Both Accelerate^®^ and ePlex^®^ testing were simultaneously started following the results of the Gram staining if the inclusion criteria were met.

### Infectious diseases consultation/Antimicrobial stewardship

A hospital-wide AMS program has been implemented at the hospital since 2011, combining treatment recommendations based on resistance data and review of and feedback on antimicrobial treatment on weekly ward rounds. Recommendations following blood culture results were not routinely performed. In this study, pathogen-related treatment recommendations were additionally implemented. After revising RDT results concerning plausibility, the ID consultant advised the attending ICU physician on therapy adaptation. Laboratory and clinical staff were trained on the intervention.

### Patient chart review

The adherence to the recommendation for AB adjustment was reviewed by AMS team members based on patient charts and microbiology results.

### Statistics

The distribution of sociodemographic and clinical variables was analyzed based on the diagnostic methods used for bloodstream infections. In the intervention group, rapid diagnostic methods (ePlex^®^ and/or Accelerate Pheno^®^) were employed, while the control group utilized conventional blood culture. Differences between the two groups were assessed under the null hypothesis (H₀) that no significant differences exist in the distribution of these variables. Appropriate statistical tests were applied: the chi-squared test for categorical (dichotomous) variables and the Wilcoxon Rank-Sum test for continuous variables that were not normally distributed, as indicated in the footnotes of **Table 1**. The normal distribution of continuous variables was determined using the Shapiro–Wilks test for normality.

**Table 1:** Patient demographics, clinical parameters, comorbidities, and outcomes.

		Standard of care group, n=44	Intervention group, n=77	p-value^a^
		n (%)	n (%)	
Basic demographics				
	Age, in years; median (IQR)	64 (58-75)	66 (59-76)	0.2
	Female, n (%)	13 (29.6)	35 (45.1)	0.09^b^
Clinical parameters on sepsis onset				
	Leukocyte count (10^9^/l), median (IQR)	15.8 (9.5-20.9)	12.8 (6.9-18.5)	0.2
	Thrombocyte count (10^9^/l), median (IQR)	199 (80-287)	131 (76-205)	0.05
	Creatinine (µmol/l), median (IQR)	135 (84-236)	152 (97-218)	0.4
	Heart frequency (beats/min), median (IQR)^c^	95 (80-124)	97 (80-111)	0.7
	Temperature (°C), median (IQR)^d^	37.5 (37.0-38.2)	37.5 (36.6-38.0)	0.4
Comorbidities				
	COPD ^c^	8 (18.2)	6 (7.9)	0.09 ^b^
	Diabetes mellitus^c^	16 (36.4)	18 (23.7)	0.1 ^b^
	Liver cirrhosis^c^	4 (9.1)	5 (6.6)	0.6 ^b^
	Malignancies^c^	4 (9.1)	15 (19.8)	0.1 ^b^
	Immune supression^c^	2 (4.6)	7 (9.2)	0.4 ^b^
	Peripheral artery disease^c^	7 (15.9)	8 (10.5)	0.4 ^b^
Clinical outcome				
	Mortality^e^	20 (45.5)	43 (56)	0.3^b^
	LOS (days), ICU, median (IQR), non-fatal cases	29.5 (13.5-37)	15.52 (6-25)	0.06
	LOS (days), ICU, median (IQR), all cases	15 (10-34.5)	12 (6-23)	0.07

IQR, interquartile range; LOS, length of stay; COPD, chronic obstructive pulmonary disease; ICU, intensive care unit.

Parameters with a high proportion of missing data were excluded from the end-analysis.

a p-value calculated using the Wilcoxson Rank-Sum test for continuous non-parametric variables, unless specified otherwise.

b p-value calculated using the chi-squared test for dichotomous variables.

c missing values, n=1 in the intervention group.

d missing values, n=3 in the intervention group.

e no statistical significance in univariate (odds ratio=1.5, 95%CI=0.6-3.0, p=0.3) and multivariate (odds ratio=1.2, 95%CI=0.6-2.7, p=0.6), using sex, thrombocyte count, and COPD as potential confounders.

We used univariable and multivariable logistic regressions to calculate the likelihood of lethal outcome using the variables sex, chronic obstructive pulmonary disease (COPD), and thrombocyte count as potential confounders as indicated by the p-values of the chi-squared test to calculate the odds ratio and the 95% confidence interval in **Table 1**. Statistical analyses were performed using STATA 17 (Stata Corp, USA). P-values for categorical values were calculated using the chi-squared test. P-values of <0.05 were considered statistically significant.

## Results

Overall, 91 patients were recruited for the intervention group and 44 for the standard of care group. In the intervention group, 82 showed a monomicrobial culture and were included in the microbiological analysis. Furthermore, 77 of these 82 (94%) patients had RDT results with either the ePlex^®^ or Accelerate PhenoTest^®^ or both (**Supplementary Figure 1B**). For 48 out of the 77 (62.3%) patients, results from both RDT systems were obtained, in contrast to 23/77 (29.9%) from the ePlex^®^ system only and 6/77 (7.8%) from the Accelerate PhenoTest^®^ only. The reasons for the unavailability of results were either that RDT was not performed due to logistics reasons (ePlex^®^ 4/82, Accelerate^®^ 14/82), that the pathogen identified in the blood culture was not included in the test panel (ePlex^®^ 3/82, Accelerate^®^ 3/82), or that the pathogen was not identified (ePlex^®^ 4/82, Accelerate^®^ 10/82). There was one incorrect species identification with the Accelerate^®^ system (coagulase-negative staphylococci instead of *S. pyogenes*). All results from the ePlex^®^ system were correct compared to the gold standard (routine cultural diagnostics). The baseline characteristics of the patients included in the analysis are summarized in **Table 1**. The species distribution identified by culture in both study arms is displayed in **Supplementary Figure 1A**.

Identification results of both RDT systems and molecular resistance markers of the ePlex^®^ system were available approximately 2 hours after the Gram stain. For species identification, both RDT systems would be suitable for delivering timely results to optimize ID/AMS consultations. While the multidrug-resistant (MDR) phenotype of staphylococci and enterococci could be reliably predicted using the detection of *van* or *mec* genes, genotypic-phenotypic AST correlation may not be accurate due to the diverse resistance mechanisms in Gram-negative rods. Furthermore, phenotypic resistance testing with the Accelerate PhenoTest^®^ was too time-consuming (>7h) to be successfully integrated into our intervention.

Thirteen patients developed BSIs caused by MDR pathogens: 10 of 77 (13.0%) in the intervention group and 3 of 44 (6.8%) in the standard-of-care group. The MDR pathogens included vancomycin-resistant *Enterococcus* (VRE), methicillin-resistant *Staphylococcus aureus* (MRSA), and third-generation cephalosporin-resistant Enterobacterales (3GCR-E). Based on the blood culture results, the initial/empirical broad-spectrum antibiotic therapy covered the pathogen spectrum in 63.6% (49/77) and 72.7% (32/44) in the intervention group and standard of care group, respectively. Adaptation to targeted antibiotic therapy was recommended in 56 of 77 cases (72.7%) based on the RDT result. De-escalation was performed in 42.8% (33/77) of the intervention group versus 22.7% (10/44) of the standard of care group. Adherence to the ID consultation postRDT results was very high, with 82.3% (50/56). In the standard of care group, an adaptation of the initial antibiotic therapy would have been recommended for 36/44 (81.8%) patients, if the species identification had been available (**Supplementary Figure 1B**). However, the adherence to local antibiotic therapy recommendation for sepsis (**Supplementary Table S1**) was significantly lower than in the intervention group (10/36; 27.8% versus 50/56; 89.3%, p<0.001, chi-squared test, H_0_=no difference between the two groups). There were no significant effects on mortality, even after adjusting for potential confounders (sex, COPD and thrombocyte count, odds ratio=1.2, 95% confidence interval 0.6-2.7, p=0.6).

## Discussion

Although the introduction of RDT in most studies leads to decreased time to effective therapy (Neuner et al., 2016), a review demonstrated that only RDT with antimicrobial stewardship programs and ID interventions decreased the mortality in both Gram-positive and Gram-negative BSIs. Although we could not show an effect on mortality due to a small number of patients with several comorbidities (**Table 1**), the length of ICU stay was reduced both in all and in non-fatal cases. Another important aspect of therapy adaptation is to reduce the resistance pressure of broad-spectrum therapy (Niederman et al., 2021). In our intervention group, de-escalation was performed more frequently than in our standard of care group. RDT combined with a subsequent ID consultation led to a reduction of the length of stay and to a better adherence to therapy recommendations and local therapy guidelines. This corresponds to the results of a large study showing significantly more frequent de-escalation following a rapid multiplex polymerase chain reaction-based blood culture identification panel combined with automatic ID consults for BSIs (Timbrook et al., 2017; Britt et al., 2023). Thus, implementation of rapid diagnostics should in general be conducted with an ID consultation to achieve the greatest benefit.

Our study has limitations. The assessment of the clinical impact of RDT/ID consultation on the mortality or clinical outcome was deemed inappropriate due to the small sample size and confounders, e.g. age >60 years and comorbidities, which significantly impact recovery and survival (Fay et al., 2020). We assume that patients with good prospects of recovery benefit most from RDT/ID consultation. However, staff availability in an ICU setting impacts timely ID consultation, a main requirement for such a study. Nonetheless, even with a relatively small sample size and simple study design, we could demonstrate that better integration of microbiological diagnostics with an ID intervention led to significantly greater adherence to antibiotic therapy recommendations and local guidelines.

Our study indicates that weekly AMS rounds and standard diagnostic stewardship alone are insufficient to ensure adequate therapy adjustments in septic patients. Combining diagnostic stewardship with an ID consultation seems to be an important requirement for high compliance with therapy recommendations for targeted therapy in septic patients.

## Data Availability

The original contributions presented in the study are included in the article/**Supplementary Material**. Further inquiries can be directed to the corresponding author.
