# Supplementary material for: Rapid diagnostic testing combined with an immediate infectious disease consultation increases the rate of septic intensive care unit patients on targeted antibiotic therapy
Source: Front Cell Infect Microbiol. 2025 Jan 21;14:1513408. doi: 10.3389/fcimb.2024.1513408 (PMC11790461; doi:10.3389/fcimb.2024.1513408)
Supplement: Supplementary file 1 [file DataSheet1.pdf]

Supplementary Table S1. Local antibiotic therapy guidelines for patients with sepsis. Abbreviations: MDRO=multidrug-resistant organisms; MRSA=methicillin-resistant *Staphylococcus aureus*.

**Sepsis**	**Antimicrobial substance**
community-acquired, infection focus unknown, no risk for MDRO	ceftriaxone
hospital-acquired, infection focus unknown, no risk for MDRO	piperacillin/tazobactam, alternative: meropenem
catheter-associated infection	vancomycin
risk factor for MRSA	vancomycin, alternative: daptomycin
**Adjustment after species identification**	**Antimicrobial substance**
Staphylococcus aureus (not colonised with MRSA)	flucloxacillin
Escherichia coli, *Klebsiella pneumoniae* (not colonised, no resistance towards 3rd generation cephalosporins)	ceftriaxone
Staphylococcus epidermidis	vancomycin
Pseudomonas aeruginosa	meropenem
Enterococcus faecalis	ampicillin
Enterococcus faecium	vancomycin
Streptoccus pneumoniae	ceftriaxone

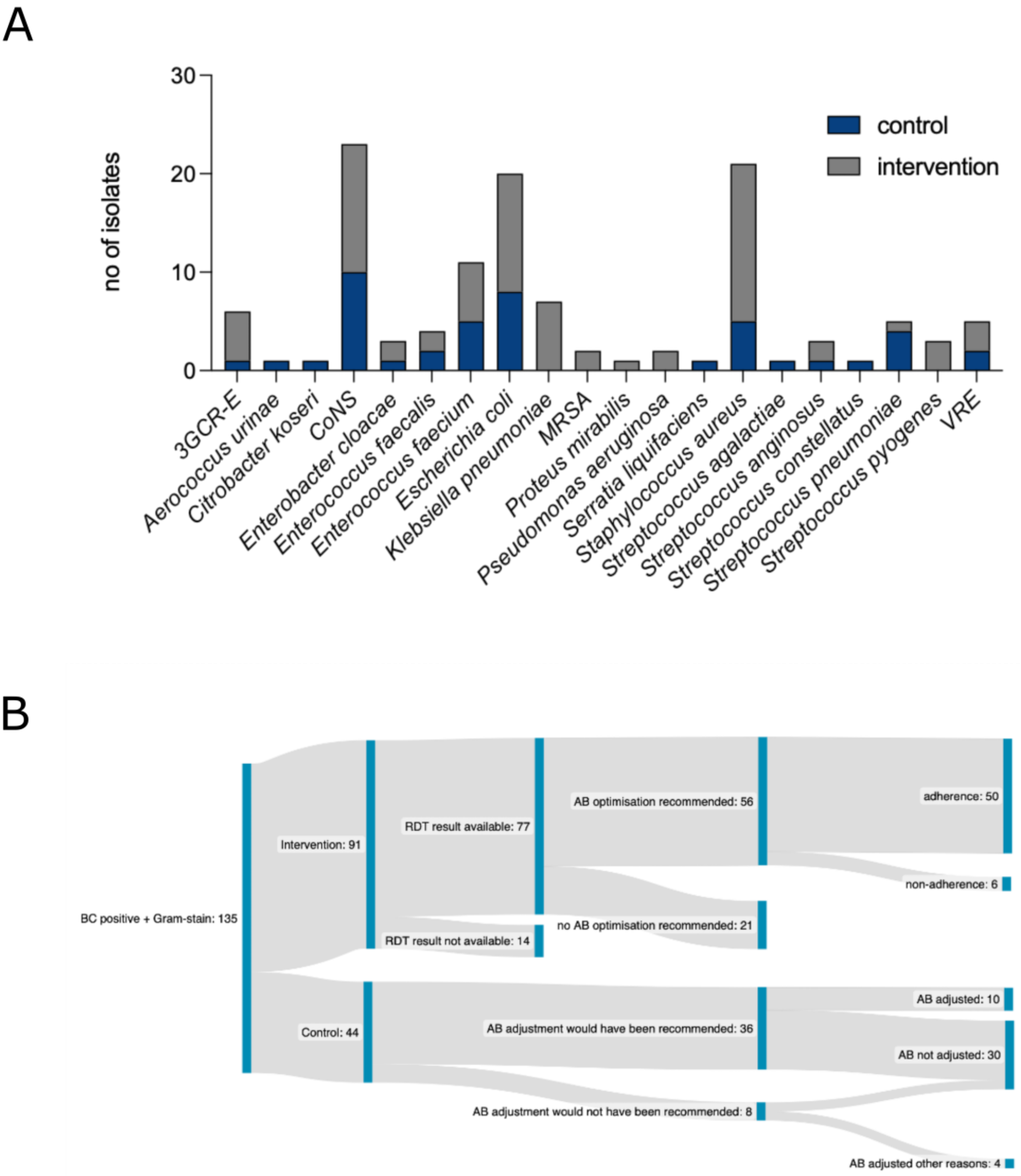

Supplementary Figure 1. Pathogen distribution and adherence to antibiotic therapy recommendations. The final results of the blood cultures for the intervention group and cont standard of care group (**A**). Overview of the rapid diagnostic test (RDT) results, subsequent antibiotic (AB) therapy adjustment recommendation and adherence to recommendations and therapy guidelines (**B**). Made with sankeyMATIC (www.sankeymatic.com). Abbreviations: BC=blood culture; CoNS=coagulase-negative staphylococci; 3GCR-E=third-generation cephalosporin-resistant Enterobacterales; VRE=vancomycin-resistant enterococci; MRSA=methicillin-resistant *Staphylococcus aureus*.
